# Presentation time shapes perceived room size in visual and auditory modalities

**DOI:** 10.1186/s41235-025-00644-3

**Published:** 2025-06-15

**Authors:** Johanna Bogon, Cindy Jagorska, Ella Maria Heinz, Martin Riemer

**Affiliations:** 1 Media Informatics Group, University of Regensburg, Regensburg, Germany; 2 Biological Psychology and Neuroergonomics, Technical University Berlin, Fasanenstr. 1, 10623 Berlin, Germany; 3 Bernstein Center for Computational Neuroscience (BCCN), Berlin, Germany

**Keywords:** Space–time interference, Space, Time, Virtual reality, Auditory, Time bisection, Task difficulty

## Abstract

Cross-dimensional interference between spatial and temporal processing provides valuable insights into the neuronal representation of space and time. Previous research has frequently found asymmetric interference patterns, with temporal judgments being more affected by spatial information than vice versa. However, this asymmetry has been attributed to the predominant use of visual paradigms (e.g., participants judge the size or duration of visual stimuli), which might facilitate spatial over temporal processing. It has been suggested that the asymmetry vanishes or even reverses when auditory stimuli are used. To test this assumption, we took advantage of the fact that acoustic reverberation carries information about the physical size of rooms. Participants judged either room size or duration, with stimuli being presented either in the visual (rooms presented in virtual reality) or the auditory modality (reverberation-based sounds). For both modalities, we found that judgments about room size were influenced by irrelevant temporal information, while judgments about duration remained unaffected by irrelevant spatial information. As time judgments were consistently rated as more difficult relative to space judgments, this pattern of interference cannot be explained on the basis of task difficulty. These results demonstrate the flexibility of space–time interference and challenge the assumption that the representation of time is necessarily based on spatial representations.

## Significance statement

Integration of spatial and temporal information is essential for many daily activities like sports (e.g., catching a ball) and navigation (e.g., crossing a busy street). Although it has consistently been found that the perception of space and time exhibits bidirectional influences (e.g., larger objects are judged as lasting longer), studies on such cross-dimensional interference between space and time are mostly restricted to abstract stimuli, such as judging the length or duration of visual 2D lines presented on a PC screen. The present study takes a more naturalistic approach by focussing on interference between the perception of temporal duration and spatial size of room interiors presented in immersive virtual reality, and by addressing the role of modality (visual versus auditory). In the real world, another important cue to estimate room size consists in the processing of acoustic reverberation (increased echo in larger rooms). The questions whether the perception of room size is influenced by irrelevant temporal information and whether perceived room size in turn has the potential to modulate temporal experience are relevant for everyday life and might have important implications, for example for interior design and architecture.

## Introduction

The way in which space and time are represented in the human brain has received a lot of interest (Eichenbaum, 2014, 2017; Ekstrom & Ranganath, 2018; Riemer et al., 2024) and cross-dimensional interference between these domains has provided valuable insights into their underlying neural mechanisms (Bonato et al., 2012; Riemer et al., 2022; Walsh, 2003). It has frequently been observed that irrelevant spatial information (e.g., the size of a stimulus) can affect temporal processing, with larger stimuli being judged as lasting longer (e.g., Casasanto & Boroditsky, 2008; Rammsayer & Verner, 2014, 2015; Starr & Brannon, 2016; Xuan et al., 2007). Also the reverse influence—where irrelevant temporal information influences spatial judgments—has been reported (Cai & Connell, 2015; Homma & Ashida, 2019; Vidaud-Laperrière et al., 2022; Whitaker et al., 2024). Such cross-dimensional interference effects have been interpreted as evidence for the existence of a neuronal system underlying the processing of general magnitudes, such as space, time, numerosity, and other dimensions (Bueti & Walsh, 2009; Riemer et al., 2022; Walsh, 2003; Winter et al., 2015). However, as in most studies the influence of time on space was less pronounced than the effect of space on time, it has been argued that this imbalance is caused by a hierarchical mental representation of temporal and spatial magnitudes. For example, Casasanto and Boroditsky (2008) proposed that the mental representation of time relies on spatial representations, which is in line with the ideas of Bergson (1888), Robbe (2023) and with the conceptual metaphor theory (Lakoff & Johnson, 1980).

In the last decade, the observation of an asymmetric interference pattern between space and time—along with the idea of a hierarchical representation of these dimensions—has been challenged by several studies demonstrating that the direction of interference depends on a variety of factors such as the modality in which the stimuli are presented (Cai & Connell, 2015; Kranjec et al., 2019), the relative difficulty of temporal versus spatial judgments (Homma & Ashida, 2015, 2019; Vidaud-Laperrière et al., 2022), and the representational noise of target and distractor dimensions (Cai & Wang, 2022; Cai et al., 2018). For a review of arguments against a genuinely asymmetric interference between time and space see Riemer and Cai (2024). One important factor driving the observation of asymmetric space–time interference seems to be the predominant use of visual paradigms (Loeffler et al., 2018; Riemer & Cai, 2024). The visual modality facilitates spatial processing, whereas the auditory modality facilitates temporal processing (Bratzke et al., 2012; Guttman et al., 2005; Handel, 1988; Ortega et al., 2014; Recanzone, 2009). There is some evidence that cross-dimensional interference is based on the relative representational acuity (e.g., Cai & Connell, 2015; Cai & Wang, 2022), in the sense that the target dimension will be affected by the interfering dimension (i) the higher the representational noise of the target dimension and (ii) the lower the representational noise of the interfering dimension (cf. Figure 1 in Riemer & Cai, 2024). Hence, assuming spatial representations to be less noisy and temporal representations to be more noisy when elicited by visual (as compared to auditory) stimuli, visual paradigms should be prone to find a relatively more pronounced effect of space on time, whereas paradigms using auditory stimuli could enhance the effect of time on space.

**Fig. 1:**
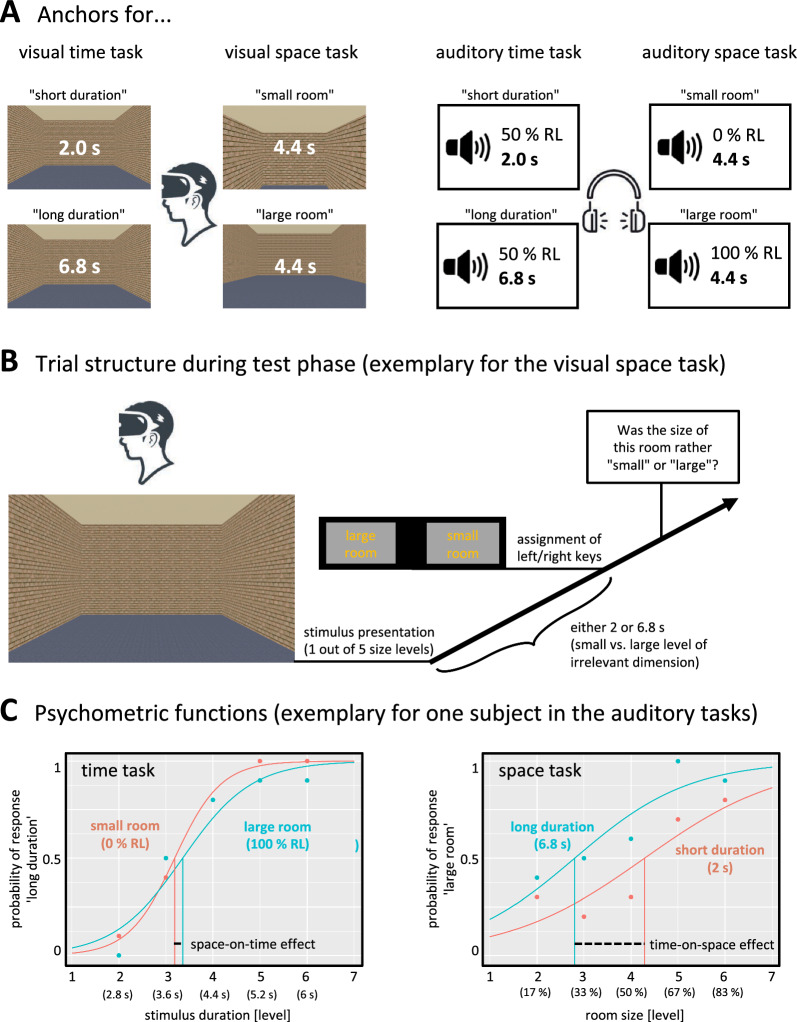
**A** Anchors for the visual and auditory bisection tasks, and **B** schematic depiction of an exemplary trial from the visual space task. Participants were presented with one out of five intermediate levels of the target dimension (linearly spaced between the two trained anchors), while the level of the irrelevant dimension was either small or large, and indicated by key press whether the target magnitude was rather small or large. Note that the meaning of keys varied across trials. **C** Quantification of interference effects, exemplary for one subject and auditory tasks. (RL = reverberation level)

One possible reason for the predominance of visual paradigms in the study of space–time interference might be the inherent imbalance in the representation of spatial and temporal magnitudes across visual and auditory experimental stimuli. While temporal magnitudes can be effectively represented in both visual and auditory modalities—since both types of stimuli can be presented for a specific duration—spatial magnitudes, such as physical size, are inherently more salient in visual stimuli. For instance, a visual square displayed on a computer screen is necessarily presented in a defined size and duration, making both magnitudes readily accessible. In contrast, auditory stimuli have clear temporal, but often lack explicit spatial magnitudes. Although sound stimuli can carry spatial information relevant for localization (e.g., Akeroyd, 2014; Amadeo et al., 2019; Dalirnaghadeh & Yilmazer, 2022; Kranjec et al., 2019), it does not inherently possess a physical size.

To test the role of modality in determining the direction of cross-dimensional interference, a paradigm is required with stimuli whose temporal and spatial magnitudes can be manipulated equally well in both visual and auditory modalities. One approach that seems well suited for this purpose is the investigation of space–time interference through judgments of room size and duration, which, to date has only been investigated with visual paradigms (DeLong, 1981; Mitchell & Davis, 1987; Riemer et al., 2018). DeLong (1981) and Mitchell and Davis (1987) presented their participants with visual model environments of different spatial scales, and Riemer et al. (2018) used 2D images of room interiors. However, there is a suitable way to transfer the spatial property of room size to the auditory domain: Due to acoustic reverberation, auditory stimuli carry information about the spatial dimensions of the surrounding environment (e.g., room size), and humans are highly sensitive to changes in reverberation levels (Kaplanis et al., 2014, 2019). This makes it possible to derive judgments about room size and duration in both the visual and the auditory modalities, providing a novel approach to quantify space–time interference across modalities.

The aim of the present study was to investigate the role of modality (visual vs. auditory) on the direction of cross-dimensional interference between the spatial size of a room and the duration for which it is presented. Room size was manipulated either visually, through differently-sized rooms presented in immersive virtual reality, or auditorily, through sounds with different levels of reverberation presented via headphones. In both modalities, participants completed a temporal bisection task, in which they judged the duration of stimulus presentation, and a spatial bisection task, in which they judged the size of the room. In both tasks, they were asked to ignore the irrelevant dimension (size or duration, respectively). We hypothesized (i) an asymmetric interference effect, with temporal judgments being more affected by spatial information than vice versa, for the visual modality, and (ii) a decrease or even a reversal of this asymmetry for the auditory modality. In accordance with previous studies (Homma & Ashida, 2015, 2019; Vidaud-Laperrière et al., 2022), we further expected that (iii) the relative difficulty of temporal and spatial judgments correlates with the direction of interference, in the sense that the difficult-to-judge dimension is more influenced by the easy-to-judge dimension.

## Methods

### Participants

Forty healthy young adults (24 females; 13 males; 3 non-binary; mean age 28 years, ranging from 22 to 45; 33 right-handed and 7 left-handed) participated in the study. They were recruited from the local community and the Technical University Berlin and received monetary compensation or course credit (their choice). Exclusion criteria consisted in hearing impairment and neurological disorders. All participants gave written informed consent to the experimental protocol, which was approved by the ethics committee of the Technical University Berlin (protocol number: MR_01_20200323). Due to extremely poor performance throughout all tasks and conditions, the data of one participant were discarded from analysis (cf. Sect. "Statistical analysis").

### Stimuli and experimental tasks

We implemented bisection tasks for time and space, and for each dimension we realized a visual and an auditory task version (Fig. 1). Visual task versions were programmed in Vizard (v.5.0, WorldViz), auditory task versions were programmed in PsychoPy v2023.1.2 (Peirce et al., 2019). Each participant performed either first both visual tasks and then both auditory tasks or vice versa. The order of time and space tasks was counterbalanced across participants, but kept constant within each participant.

#### Visual bisection tasks

In the visual time bisection task, participants sat on a chair in the tracking area of the Vive camera system. They wore a head-mounted display (HTC Vive Pro) and were presented with virtual environments (rooms of different sizes, presented for different durations). Except for the instruction to remain seated and to maintain their general body orientation, they were allowed to move, rotate their head and look around.

The task was composed of a training phase, in which participants were familiarized with a “short” (2.0 s) and a “long” (6.8 s) standard duration, and a test phase, in which they had to categorize five linearly spaced intermediate test durations (2.8, 3.6, 4.4, 5.2, or 6.0 s) as either “short” or “long” (unspeeded two-alternative forced choice).

During the training phase, participants performed four reproduction trials,^1^ then eight bisection trials, and finally again four reproduction trials (within these trial types, the order of short and long standards was randomized). In each training trial, either the short or the long standard was presented and the participants had to terminate a second interval by pressing the space bar as soon as it had reached the same duration as the standard (reproduction trial), or they had to decide whether it was the short or the long standard (bisection trial). In the training phase, standard durations were indicated by the presentation of a medium-sized room (10 m width, 15 m length, 6 m height).

During the test phase, in each trial one test duration was presented and participants had to indicate whether this duration was more equal to the short or to the long standard. Unspeeded responses were given with the left or the right key. To increase the comparability between time and space tasks by introducing a task-independent forced waiting period before choosing the correct key, and to counteract preexisting associations between the spatial arrangement of response keys (e.g., left/right) and temporal or spatial factors (e.g., short/long or small/large; Anelli & Frassinetti, 2019; Bogon et al., 2024; Bonato et al., 2012), the assignment of the response keys to the categories “short” and “long” varied between trials and was indicated after the offset of the test duration. Test durations were indicated either by the presentation of a small room (4 m width, 6 m length, 3 m height) or a large room (16 m width, 24 m length, 9 m height). The test phase comprised 100 trials, that is, each of the five test durations was presented 10 times with a small and 10 times with a large room (randomized order).

Participants were instructed to refrain from chronometric counting.

In the visual space bisection task, participants learned in the training phase a small (4 m width, 6 m length, 3 m height) and a large virtual room (16 m width, 24 m length, 9 m height) as standards, and in the test phase they judged whether intermediate room sizes (five equidistant steps between the small and the large standard for width, length, and height) were more equal to the small or the large standard room size. Virtual rooms were presented either for a short (2.0 s) or a long duration (6.8 s). The only difference to the visual time bisection task was related to the training procedure, where no reproduction trials and instead 16 bisection trials were presented. All other aspects of the visual space bisection task were analogous to the visual time bisection task.

After completion of both visual bisection tasks, participants rated on a visual analogue scale the relative task difficulty (from “space task was more difficult” to “time task was more difficult”).

#### Auditory bisection tasks

Except for the use of acoustic stimuli, the auditory versions of time and space bisection tasks were analogous to the visual task versions. Auditory stimuli were sound files of five elongated vowels (sustained phonation of /a/, /e/, /i/, /o/, /u/) spoken by a female human, presented binaurally via headphones (Stereo TW-260A). Sound files were edited with Audacity 3.2.5 and overlaid with seven different preverb levels simulating different room sizes in equidistant steps from 0 and 100% reverberation (0, 17, 33, 50, 67, 83, 100%). As the physically correct reverberation level for a room of specific size depends on many other factors (e.g., material of walls, distance to walls, location of sound source), reverberation levels were not matched to the visual room sizes used in the visual bisection tasks (Sect. "Visual bisection tasks").

In the auditory time bisection task, five intermediate test durations (2.8, 3.6, 4.4, 5.2, or 6.0 s) were presented with either the smallest or the largest reverberation level (0 or 100%). And in the auditory space bisection task, the five intermediate reverberation levels (17, 33, 50, 67, or 83%) were presented with either the shortest or the longest duration (2 or 6.8 s). All other aspects (trial numbers, training procedure, etc.) were as described for the visual task versions. A difference was that participants sat in front of a PC screen and wore headphones (instead of a head-mounted display). In the training phase of the auditory space bisection task, the two standards were referred to as “a sound in a very small room” and “a sound in a very large room”. After completion of both auditory bisection tasks, participants rated relative task difficulty as described above for the visual tasks (cf. Sect. "Visual bisection tasks").

### Statistical analysis

For each participant and each of the four tasks (time and space judgments in two modalities each), two psychometric functions were calculated, describing the performance depending on the level of the irrelevant dimension (Fig. 1C). Responses later than 15 s or earlier than 200 ms were discarded as outliers (0.1% of all trials). Fitted logistic functions were calculated using R package *quickpsy* (Linares & López-Moliner, 2016), and represent the probability of categorizing the respective test stimulus as “more similar to the longer/larger” standard as a function of the relevant stimulus magnitude. To ensure a comparable metric between time and space tasks, relevant stimulus magnitude was quantified by the number of the five intermediate steps (i.e., numerical values from 2 to 6). Guess and lapse rates were allowed to vary between 0 and 0.1. From the resulting functions we extracted the point of subjective equality (PSE), defined as the value of the x-axis corresponding to 50% on the y-axis, and the difference limen (DL), defined as half the difference between the values of the x-axis corresponding to 25% and 75% on the y-axis (i.e., the flatter the logistic function, the larger the DL; Ulrich & Vorberg, 2009). Functions with a DL above 6 (indicating poor general performance) or an extreme PSE outside the tested magnitude range were defined as outliers and discarded from further analysis (according to this procedure, 2.8% of functions were discarded). Interference effects were quantified by the PSE difference between the small and the large level of the irrelevant dimension (cf. Figure 1C). Judgment precision (an indicator for task difficulty) was quantified by the mean DL across both levels of the irrelevant condition.

For the analysis of reaction times, additional outliers were defined according to the Median Absolute Deviation procedure (MAD; Leys et al., 2013), performed within each participant, target dimension and modality, and with a cutoff value of 10. In total, 2.7% of reaction time data were discarded as outliers.

Data were analyzed in R (R Core Team, 2016) by fitting linear mixed-effects models (2 × 2 factorial design) using packages *lme4* (Bates et al., 2015) and *lmerTest* (Kuznetsova et al., 2017), including the within-subjects factors *target dimension* (time vs. space) and *modality* (visual vs. auditory). Subjects were included as random factor. Analysis script and raw data can be found at OSF (https://osf.io/v9wxn/).

## Results

**Fig. 2:**
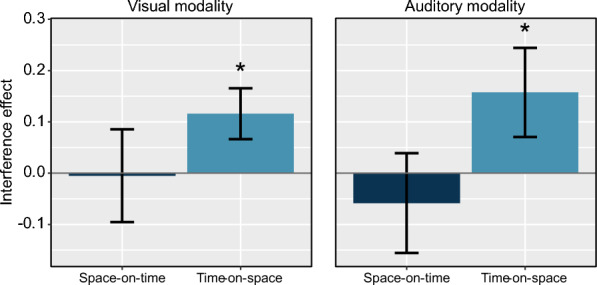
Interference effects in the temporal bisection task (dark blue bars) and the spatial bisection task (light blue bars) for the visual and auditory modality. Interference effects are quantified by the PSE difference between the small and the large level of the irrelevant dimension (see Sect. "Statistical analysis"). Error bars represent the standard error across participants. (* *p* < .05)

Interference effects are depicted in Fig. 2. We found a significant main effect of *target dimension* (β = −0.085, SE = 0.040, t_113_ = −2.1, *p* = 0.038), whereas the main effect of *modality* (β = −0.002, SE = 0.040, t_113_ = −0.1, *p* > 0.5) and the respective interaction effect (β = −0.026, SE = 0.040, t_113_ = −0.6, *p* > 0.5) did not reach a significant level. As can be seen in Fig. 2, this pattern of results indicates that, for both the visual and the auditory modality, space judgments were more influenced by irrelevant temporal information than vice versa. One-tailed t-tests confirmed the effects of time on space (visual: t_38_ = 2.3, *p* = 0.015; auditory: t_37_ = 1.8, *p* = 0.038), whereas the effects of space on time were not significantly different from zero (visual: |t_38_|< 0.1, *p* > 0.5; auditory: t_38_ = −0.6, *p* > 0.5).

**Fig. 3:**
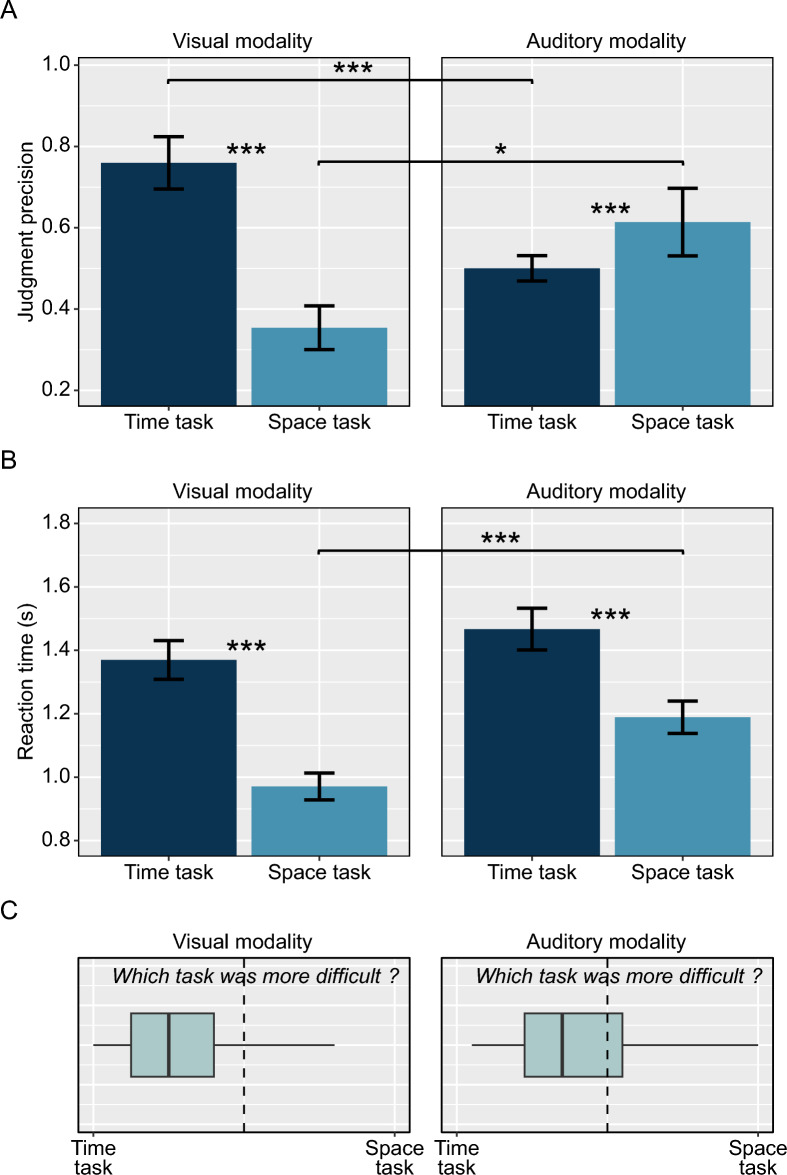
**A** Judgment precision and **B** reaction times in the temporal bisection task (dark blue bars) and the spatial bisection task (light blue bars) in the visual and auditory modality. Judgment precision was quantified by the mean DL across both levels of the irrelevant condition, so that smaller values indicate higher precision (see Sect. "Statistical analysis"). Error bars represent the standard error across participants. **C** Subjective ratings of relative task difficulty. (* *p* < .05; *** *p* < .001)

With respect to task difficulty (Fig. 3A), the analysis of judgment precision revealed a significant main effect of *target dimension* (β = 0.071, SE = 0.028, t_114_ = 2.5, *p* = 0.014) indicating that the time task was more difficult than the space task, and a significant interaction effect between *target dimension* and *modality* (β = −0.131, SE = 0.028, t_114_ = −4.6, *p* < 0.001) indicates that this advantage of the space task was driven by the visual modality. There was no main effect of *modality* (β = 0.001, SE = 0.028, t_114_ = 0.1, *p* > 0.5). Subsequent t-tests revealed that time judgments are more precise in the auditory as compared to the visual modality (t_38_ = −3.8, *p* < 0.001), whereas space judgments are more precise in the visual as compared to the auditory modality (t_38_ = 2.6, *p* = 0.012).

For reaction times (Fig. 3B), we found significant main effects of *target dimension* (β = 0.169, SE = 0.019, t_114_ = 8.9, *p* < 0.001), indicating faster responses in the space tasks, and of *modality* (β = 0.079, SE = 0.019, t_114_ = 4.1, *p* < 0.001), indicating faster responses for the visual modality. There was no significant interaction effect between *target dimension* and *modality* (β = −0.030, SE = 0.019, t_114_ = −1.6, *p* = 0.12).

In spite of the direction of cross-dimensional interference (space being more affected by irrelevant temporal information than vice versa), the subjective ratings (Fig. 3C) revealed that the time task was perceived as more difficult than the space task, both for the visual (t_38_ = 6.1, *p* < 0.001) and the auditory modality (t_38_ = 2.4, *p* = 0.024). No correlations were found between difficulty ratings and interference effects (all *p* > 0.25).

## Discussion

In the present study we investigated cross-dimensional interference between the perception of spatial room size and temporal duration, as well as how these interference effects are modulated by the sensory modality in which the temporal and spatial information is presented. As the representation of room size can be assumed to be less precise when spatial information is provided only via the auditory modality (i.e., via the reverberation level of sounds) and the representation of temporal duration is known to be more precise for the auditory modality (Bratzke et al., 2012; Guttman et al., 2005; Handel, 1988; Ortega et al., 2014), we expected that the direction of interference depends on the modality. Specifically, we expected that temporal judgments are more susceptible to spatial interference in the visual modality, while spatial judgments are more susceptible to temporal interference in the auditory modality.

The main (and unexpected) finding is that irrelevant temporal information exerted a greater influence on judgments of spatial room size, in both the visual and the auditory modality (Fig. 2). In terms of the bisection paradigm implemented here, visual and auditory stimuli were more likely to be associated with a large room, when they were presented for a longer duration. In contrast, no such bias was found for duration judgments on stimuli containing (irrelevant) information about room size. With respect to the auditory domain, this direction of space–time interference is in line with the idea that the direction of cross-dimensional interference depends on the relative representational noise (Cai & Connell, 2015; Cai & Wang, 2022; Riemer & Cai, 2024): The processing of auditory information to infer room size coincides with a higher level of noise (compared to visual information about room size), and therefore it is more affected by the irrelevant temporal magnitude. However, with respect to the visual dimension, it is difficult to reconcile the larger time-on-space effect with the same framework.

In other studies demonstrating a more pronounced effect of time on space (relative to the effect of space on time), this is usually explained by the relative difficulty of spatial and temporal judgments, according to the logic that the relatively easy-to-judge dimension influences the relatively difficult dimension to a larger extent than vice versa (Cai & Connell, 2015; Homma & Ashida, 2019; Vidaud-Laperrière et al., 2022). However, the results of the present study are inconsistent with this explanation. Independent of modality, the time task was consistently rated as more difficult than the space task (Fig. 3C). Moreover, these ratings of relative task difficulty did not correlate with the individual degree of space–time interference. In addition, spatial judgments of visual stimuli were much more precise and executed faster than temporal judgments, which also implies a higher difficulty of the time task, even using more implicit behavioral parameters. At least for the visual modality, it is unlikely that the larger time-on-space effect is caused by temporal judgments being easier (and therefore less noisy) than spatial judgments about room size.

Another potential explanation relates to the lower attentional demands of the bisection method implemented in the present study, which deviates from many other studies on space–time interference mostly using the methods of reproduction and discrimination (cf. Table 1 in Riemer & Cai, 2024). The bisection tasks implemented here are less demanding with respect to attentional resources in two ways. First, in every trial, the target dimension was already known while the stimuli were presented. This contrasts with many other studies, in which the target dimension was indicated only after the offset of the stimulus, requiring the the participants to divide their attention between time and space information (e.g., Cai & Wang, 2022; Merritt et al., 2010; Whitaker et al., 2024). It seems plausible that the simultaneous attention to two different stimulus features promotes interference between those features.^2^ Second, we used a block design, that is, time and space judgments did not alternate unexpectedly between trials, so that there was no need to shift the focus of attention on a trial-by-trial basis.

In bisection tasks, the critical stimuli are those with the highest degree of uncertainty (usually the ones lying exactly in the middle between the two anchors). For uncertain space trials, this state of uncertainty is reached immediately after stimulus onset, because all the information needed to decide whether the room is rather small or large is immediately present. But the stimulus presentation continues, and as the magnitude of the target dimension has already been processed as “uncertain”, attentional resources are free for processing the irrelevant information about time. In contrast, for uncertain time trials the state of uncertainty is reached only when the stimulus ends after an intermediate duration which is difficult to categorize as short or long. At this point, when the magnitude of the target dimension is considered “uncertain”, the irrelevant spatial information is not present anymore and cannot be attended to. As a consequence, task-irrelevant temporal information might exert a greater influence on spatial judgments than vice versa.

This post-hoc explanation of our results is specific to comparative judgments (e.g., bisection or discrimination tasks) and non-accumulating spatial stimuli. It does not apply to reproduction tasks, because in this method the trial is terminated by the participant when the highest degree of uncertainty is reached (Boned & López-Moliner, 2024; Riemer et al., 2016), preventing any further influence on the judgment. It also does not apply to study designs using accumulating spatial stimuli, such as growing length (e.g., Dormal & Pesenti, 2013; Lambrechts et al., 2013; Vidaud-Laperrière et al., 2022), because in these cases the complete information about the spatial magnitude is only available at the end of stimulus presentation (analogous to the temporal magnitude). We found three studies on space–time interference implementing bisection tasks with non-accumulating spatial stimuli (Homma & Ashida, 2015, 2019; Merritt et al., 2010). Of those, only the two studies by Homma and Ashida (2015, 2019) used a design comparable to the one presented here. In Merritt et al. (2010), participants had to simultaneously attend to both space and time, which interferes with the proposed redirection of attention towards the irrelevant dimension after the magnitude of the target dimension is considered “uncertain”, because the target dimension was unknown to the participants until stimulus offset.^3^ The hypothesis outlined above is in line with Homma and Ashida (2019), who also found the time-on-space effect to be larger than the space-on-time effect, but it does not align with the findings of Homma and Ashida (2015), who observed a larger space-on-time effect. However, it should be noted that in the experiment in which Homma and Ashida (2015) found a larger space-on-time effect, relatively short durations (400–800 ms) were used, which may have limited the time available to redirect attention after the spatial magnitude has been processed. When the range of tested durations was increased to 1000–4000 ms in the same experimental design, a larger time-on-space effect was observed (Homma & Ashida, 2019).

The idea that cross-dimensional influences are modulated by the type of the experimental paradigm used is not new. For example, Yates et al. (2012) found the space-on-time effect reported by Xuan et al., (2007; i.e., that larger stimuli are perceived to last longer) to be reversed when probed with equality judgments (e.g., *Are two successively presented stimuli equal or different in duration?*) instead of comparative judgments (e.g., *Which one of two stimuli was longer?*). As the implementation of equality judgments reduces the influence of a potential response bias, Yates et al. (2012) argue that space–time interference effects are rather a matter of biased decisions than of a genuine change in perception. In other words, larger stimuli are not *perceived* as lasting longer, they are only *judged* as lasting longer. According to this account, it is possible that the bisection task implemented here (as an instance of comparative judgments) facilitated the influence of response biases and that this unproportionally supported the time-on-space effect, according to the rationale of our post-hoc explanation described above.

The present finding of a larger time-on-space effect (both for visual and auditory stimuli) stands in contrast to the idea of a fixed hierarchical representation of spatial and temporal magnitudes as proposed by Casasanto and Boroditsky (2008). Instead, it demonstrates that the direction of interference between space and time is more flexible and may depend on various factors such as the paradigm implemented to judge spatial and temporal magnitudes, in addition to the factors of task difficulty (Homma & Ashida, 2015, 2019; Vidaud-Laperrière et al., 2022), representational noise (Cai & Wang, 2022; Cai et al., 2018), and the predominant use of visual paradigms (Kranjec et al., 2019; Loeffler et al., 2018). This interpretation is in line with recent studies showing a symmetric pattern of space–time interference (Whitaker et al., 2024) and strengthens the view that the asymmetric interaction patterns observed in previous studies on space–time interference are the result of methodological aspects rather than by a genuine dependency of temporal on spatial representations (Riemer & Cai, 2024).

## Conclusions

We investigated how sensory modality influences the direction of cross-dimensional interference between spatial and temporal information. The results demonstrate that cross-dimensional interference between space and time is more flexible than previously assumed. Even in a paradigm using visual stimuli, spatial representations can sometimes be more influenced by temporal representations than vice versa. Furthermore, the direction of interference does not seem to be entirely determined by relative task difficulty. Especially for the bisection method and non-accumulating spatial stimuli, the role of attentional demands (and the time course of directed attention to either dimension) is a promising factor and should be tested in future studies. Our findings challenge the assumption of a fixed hierarchical relationship between space and time, and showcase the important role of task design, attentional demands, and representational noise in shaping the direction of space–time interference.

## Data Availability

The data and materials from the present experiment are publicly available at the Open Science Framework website: https://osf.io/v9wxn/.

## Footnotes

1 The implementation of reproduction trials in the training phase of the time tasks ensured that participants fully processed the „long “ standard duration (rather than just learning to differentiate between „short “ and „not short “).

2 Note, however, that in Experiments 2–6 by Casasanto and Boroditsky (2008) the target dimension was also announced before stimulus presentation and still an asymmetric interference favoring the space-on-time effect was found.

3 Note that in Experiment 2a of Merritt et al. (2010), the target dimension was indicated before stimulus processing, but the participants in this experiment were two rhesus monkeys.
